# Supplementary material for: Fatty Liver and Risk of Head and Neck Cancer in Type 2 Diabetes Mellitus: A Nationwide Cohort Study
Source: Cancers (Basel). 2023 Feb 14;15(4):1209. doi: 10.3390/cancers15041209 (PMC9954043; doi:10.3390/cancers15041209)
Supplement: Supplementary file 1 [file cancers-15-01209-s001.zip › cancers-2169920-supplementary.pdf]

# Supplementary Online Content

## Supplementary materials

Supplementary Table S1. General characteristics of the subjects according to the risk of Head and Neck Cancer among DM patients.

Supplementary Table S2. Association between components of fatty liver index and risk of head and neck cancer in DM patients.

This supplementary material has been provided by the authors to give readers additional information about their work

Supplementary Table S1. General characteristics of the subjects according to the risk of head and neck cancer among type 2 diabetes mellitus patients.

| Variables | Any Head and Neck Cancer | | *p*-value |
|---|---|---|---|
| | No | Yes | |
| Total, n (%) | 1,901,258 (99.8) | 3,118 (0.2) | |
| Age, years, mean ± SD | 57.6 ± 12.5 | 62.8 ± 10.0 | <0.001 |
| Age, years | | | <0.001 |
| <30 y | 28,786 (1.5) | 3 (0.1) | |
| 30 ~ <40 y | 121,239 (6.4) | 42 (1.4) | |
| 40 ~ <50 y | 336,969 (17.7) | 246 (7.9) | |
| 50 ~ <60 y | 535,297 (28.2) | 815 (26.1) | |
| 60 ~ <70 y | 507,174 (26.7) | 1,140 (36.6) | |
| 70 ~ <80 y | 315,823 (16.6) | 755 (24.2) | |
| ≥ 80 y | 55,970 (2.9) | 117 (3.8) | |
| Sex (male) | 1,070,160 (56.3) | 2,501 (80.2) | <0.001 |
| Income (lowest quartile) | 447,219 (23.5) | 753 (24.2) | 0.409 |
| Smoking status | | | <0.001 |
| Never smoker | 1,133,225 (59.6) | 1,281 (41.1) | |
| Ex-smoker | 32,2278 (17.0) | 735 (23.6) | |
| Current smoker | 445,755 (23.5) | 1,102 (35.3) | |
| Smoking status | | | <0.001 |
| Never smoker | 1,133,225 (59.6) | 1,281 (41.1) | |
| Ex-smoker, < 20 PY | 176,594 (9.3) | 308 (9.9) | |
| Ex-smoker, ≥ 20 PY | 145,684 (7.7) | 427 (13.7) | |
| Current smoker, < 20 PY | 219,761 (11.6) | 340 (10.9) | |
| Current smoker, ≥ 20 PY | 225,994 (11.9) | 762 (24.4) | |
| Smoking, PY | | | <0.001 |
| Non-smoking | 1,133,225 (59.6) | 1,281 (41.1) | |
| <5 PY | 103,700 (5.5) | 132 (4.2) | |
| 5 ~ <10 PY | 111,549 (5.9) | 195 (6.3) | |
| 10 ~ <15 PY | 97,056 (5.1) | 206 (6.6) | |
| 15 ~ <20 PY | 371,678 (19.6) | 1,189 (38.1) | |
| ≥20 PY | 84,050 (4.4) | 115 (3.7) | |
| Alcohol consumption | | | <0.001 |
| Non-drinker | 1,189,761 (62.6) | 1,710 (54.8) | |
| Mild drinker | 711,497 (37.4) | 1,408 (45.2) | |
| Physical activity. regular | 385,559 (20.3) | 697 (22.4) | 0.004 |
| Height (cm) | 161.8 ± 9.4 | 164.0 ± 7.9 | <0.001 |
| Weight (kg) | 65.8 ± 11.8 | 66.2 ± 10.5 | 0.112 |
| Waist Circumference (cm) | 85.1 ± 8.7 | 86.3 ± 8.2 | <0.001 |
| BMI, mean, $kg/m^2$ | 25.1 ± 3.4 | 24.6 ± 3.2 | <0.001 |
| BMI, $kg/m^2$ | | | <0.001 |
| <18.5 | 29,519 (1.6) | 74 (2.4) | |
| 18.5 ~ <23 | 476,772 (25.1) | 869 (27.9) | |
| 23 ~ <25 | 474,940 (25.0) | 865 (27.7) | |
| 25 ~ <30 | 774,033 (40.7) | 1,154 (37.0) | |
| ≥30 | 145,994 (7.7) | 156 (5.0) | |
| Hypertension | 1,062,940 (55.9) | 1,993 (63.9) | <0.001 |
| Dyslipidemia | 799,961 (42.1) | 1,257 (40.3) | 0.047 |
| DM (≥5 years) | 579,379 (30.5) | 1,131 (36.3) | <0.001 |
| Insulin | 141,188 (7.4) | 264 (8.5) | 0.027 |
| OHA (≥ 3 drugs) | 253,334 (13.3) | 513 (16.5) | <0.001 |
| SBP, mmHg | 128.9 ± 15.9 | 130.8 ± 16.2 | <0.001 |
| DBP, mmHg | 78.9 ± 10.2 | 79.1 ± 10.4 | 0.331 |
| Glucose, mg/dL | 144.7 ± 46.8 | 141.5 ± 45.2 | <0.001 |
| GFR, $mL/min/1.73m^2$ | 84.5 ± 35.7 | 82.5 ± 40.9 | 0.003 |
| Total cholesterol, mg/Dl | 197.7 ± 42.4 | 192.9 ± 41.9 | <0.001 |
| HDL, mg/dL | 51.6 ± 21.8 | 51.1 ± 24.6 | 0.167 |
| LDL, mg/dL | 113.2 ± 40.7 | 109.4 ± 45.0 | <0.001 |
| *TG, mg/dL | 144.3 (144.2–144.4) | 144.8 (141.9–147.7) | 0.758 |

* Values presented as Geometric mean (95% Confidence Interval)

FLI, fatty liver index; PY, pack-years; BMI, body mass index; DM, diabetes mellitus; OHA, oral hypoglycemic agent; SBP, Systolic Blood Pressure; DBP, Diastolic Blood Pressure ;GFR, glomerular filtration rate; HDL, high density lipoprotein cholesterol; LDL, low density lipoprotein cholesterol; TG, triglyceride.

Supplementary Table S2. Association between components of fatty liver index and risk of head and neck cancer in type 2 diabetes mellitus patients.

| Cancer subtypes | BMI (kg/$m^2$) | Number of subjects (%) | Cases | Duration (Person-years) | IR | Model 1 aHR[1] (95% CI) | Model 2 aHR[2] (95% CI) | Model 3 aHR[3] (95% CI) |
|---|---|---|---|---|---|---|---|---|
| Oral cavity | < 25 | 983,039 (51.6) | 509 | 6,738,004 | 7.55 | 1 (Ref.) | 1 (Ref.) | 1 (Ref.) |
| | ≥ 25 | 921,337 (48.4) | 440 | 6,428,065 | 6.85 | 0.91 (0.74–1.11) | 0.91 (0.74–1.11) | 0.91 (0.75–1.11) |
| Pharynx | < 25 | 983,039 (51.6) | 684 | 6,737,629 | 10.15 | 1 (Ref.) | 1 (Ref.) | 1 (Ref.) |
| | ≥ 25 | 921,337 (48.4) | 468 | 6,427,943 | 7.28 | 1.04 (0.86–1.26) | 1.04 (0.86–1.25) | 1.04 (0.86–1.25) |
| Larynx | < 25 | 983,039 (51.6) | 688 | 6,737,326 | 10.21 | 1 (Ref.) | 1 (Ref.) | 1 (Ref.) |
| | ≥ 25 | 921,337 (48.4) | 397 | 6,428,003 | 6.18 | 0.88 (0.72–1.06) | 0.87 (0.72–1.06) | 0.87 (0.72–1.06) |
| Salivary gland | < 25 | 983,039 (51.6) | 180 | 6,738,690 | 2.67 | 1 (Ref.) | 1 (Ref.) | 1 (Ref.) |
| | ≥ 25 | 921,337 (48.4) | 177 | 6,428,701 | 2.75 | 1.08 (0.78–1.50) | 1.09 (0.79–1.51) | 1.10 (0.79–1.52) |
| Cancer subtypes | TG (mg/dL) | | | | | | | |
| Oral cavity | < 150 | 1,024,618 (53.8) | 506 | 7,064,351 | 7.16 | 1 (Ref.) | 1 (Ref.) | 1 (Ref.) |
| | ≥ 150 | 879,758 (46.2) | 443 | 6,101,719 | 7.26 | 1.09 (0.96–1.24) | 1.05 (0.92–1.19) | 1.06 (0.93–1.20) |
| Pharynx | < 150 | 1,024,618 (53.8) | 606 | 7,064,114 | 8.58 | 1 (Ref.) | 1 (Ref.) | 1 (Ref.) |
| | ≥ 150 | 879,758 (46.2) | 546 | 6,101,459 | 8.95 | 1.15 (1.02–1.30) | 1.11 (0.98–1.25) | 1.12 (0.99–1.26) |
| Larynx | < 150 | 1,024,618 (53.8) | 571 | 7,063,902 | 8.08 | 1 (Ref.) | 1 (Ref.) | 1 (Ref.) |
| | ≥ 150 | 879,758 (46.2) | 514 | 6,101,427 | 8.42 | 1.22 (1.08–1.38) | 1.13 (1.00–1.28) | **1.14 (1.01–1.29)** |
| Salivary gland | < 150 | 1,024,618 (53.8) | 189 | 7,064,995 | 2.68 | 1 (Ref.) | 1 (Ref.) | 1 (Ref.) |
| | ≥ 150 | 879,758 (46.2) | 168 | 6,102,396 | 2.75 | 1.05 (0.85–1.30) | 1.05 (0.85–1.30) | 1.07 (0.87–1.33) |
| Cancer subtypes | WC (cm) | | | | | | | |
| Oral cavity | M<90,W<80 | 990,173 (51.2) | 467 | 6,815,946 | 6.85 | 1 (Ref.) | 1 (Ref.) | 1 (Ref.) |
| | M≥90,W≥80 | 914,203 (48.0) | 482 | 6,350,124 | 7.59 | **1.21 (1.02–1.42)** | **1.19 (1.00–1.40)** | 1.18 (1.00–1.39) |
| Pharynx | M<90,W<80 | 990,173 (51.2) | 705 | 6,815,372 | 10.34 | 1 (Ref.) | 1 (Ref.) | 1 (Ref.) |
| | M≥90,W≥80 | 914,203 (48.0) | 447 | 6,350,201 | 7.04 | 1.16 (1.00–1.36) | 1.14 (0.98–1.33) | 1.14 (0.98–1.33) |
| Larynx | M<90,W<80 | 990,173 (51.2) | 705 | 6,815,139 | 10.35 | 1 (Ref.) | 1 (Ref.) | 1 (Ref.) |
| | M≥90,W≥80 | 914,203 (48.0) | 380 | 6,350,190 | 5.98 | 1.12 (0.95–1.31) | 1.07 (0.91–1.26) | 1.07 (0.91–1.25) |
| Salivary gland | M<90,W<80 | 990,173 (51.2) | 175 | 6,816,594 | 2.57 | 1 (Ref.) | 1 (Ref.) | 1 (Ref.) |
| | M≥90,W≥80 | 914,203 (48.0) | 182 | 6,350,796 | 2.87 | 1.28 (0.97–1.68) | 1.27 (0.97–1.67) | 1.27 (0.96–1.66) |
| Cancer subtypes | GGT (IU/L) | | | | | | | |
| Oral cavity | M<63,W<35 | 1,436,534 (75.4) | 682 | 9,949,374 | 6.85 | 1 (Ref.) | 1 (Ref.) | 1 (Ref.) |
| | M≥63,W≥35 | 467,842 (24.6) | 267 | 3,216,695 | 8.30 | **1.48 (1.28–1.71)** | **1.41 (1.22–1.63)** | **1.44 (1.24–1.68)** |
| Pharynx | M<63,W<35 | 1,436,534 (75.4) | 850 | 9,948,896 | 8.54 | 1 (Ref.) | 1 (Ref.) | 1 (Ref.) |
| | M≥63,W≥35 | 467,842 (24.6) | 302 | 3,216,676 | 9.39 | **1.39 (1.21–1.59)** | **1.33 (1.16–1.53)** | **1.34 (1.17–1.54)** |
| Larynx | M<63,W<35 | 1,436,534 (75.4) | 793 | 9,948,717 | 7.97 | 1 (Ref.) | 1 (Ref.) | 1 (Ref.) |
| | M≥63,W≥35 | 467,842 (24.6) | 292 | 3,216,613 | 9.08 | **1.58 (1.37–1.81)** | **1.47 (1.28–1.70)** | **1.50 (1.30–1.73)** |
| Salivary gland | M<63,W<35 | 1,436,534 (75.4) | 290 | 9,950,222 | 2.91 | 1 (Ref.) | 1 (Ref.) | 1 (Ref.) |

| | | | | | | | |
|---|---|---|---|---|---|---|---|
| M≥63,W≥35 | 467,842 (24.6) | 67 | 3,217,168 | 2.08 | 0.78 (0.60–1.02) | 0.80 (0.61–1.06) | 0.83 (0.63–1.10) |

M, male; F, female; BMI, body mass index; WC, waist circumference; TG, triglyceride. GGT, gamma-glutamyl-transferase; IR, incidence rate (per 100,000 person-years); HR, hazard ratio; aHR, adjusted hazard ratio; CI, confidence interval.

[1]Adjusted for age, sex, and BMI.

[2]Adjusted for age, sex, BMI, smoking, alcohol consumption, regular physical activity, and income.

[3]Adjusted for age, sex, BMI, smoking, alcohol consumption, regular physical activity, income, glucose, insulin, oral hypoglycemic agent, and diabetes mellitus duration.

Statistically significant values are marked in bold.
